# Pharmacokinetics and Tissue Levels of Pantoprazole in Neonatal Calves After Intravenous Administration

**DOI:** 10.3389/fvets.2020.580735

**Published:** 2020-11-27

**Authors:** Jeff D. Olivarez, Amanda J. Kreuder, Dane M. Tatarniuk, Larry W. Wulf, Katarzyna A. Dembek, Jonathan P. Mochel, Joe S. Smith

**Affiliations:** ^1^Lloyd Veterinary Medical Center, College of Veterinary Medicine, Iowa State University, Ames, IA, United States; ^2^Veterinary Microbiology and Preventive Medicine, College of Veterinary Medicine, Iowa State University, Ames, IA, United States; ^3^Veterinary Clinical Sciences, College of Veterinary Medicine, Iowa State University, Ames, IA, United States; ^4^Analytical Chemistry Services, College of Veterinary Medicine, Iowa State University, Ames, IA, United States; ^5^Veterinary Diagnostic and Production Animal Medicine, College of Veterinary Medicine, Iowa State University, Ames, IA, United States; ^6^Biomedical Sciences, College of Veterinary Medicine, Iowa State University, Ames, IA, United States

**Keywords:** calf, bovine, pantoprazole, pharmacokinetics, tissue residue, pantoprazole sulfone

## Abstract

**Background:** Neonatal calves are at risk of developing abomasal ulceration, but there is a lack of pharmacokinetic data for potential anti-ulcerative therapies, such as pantoprazole, in ruminant species.

**Objective:** The study objectives were to estimate plasma pharmacokinetic parameters for pantoprazole in neonatal dairy calves after intravenous (IV) administration. A secondary objective was to quantify the concentrations of pantoprazole in edible tissues after IV dosing.

**Methods:** Pantoprazole was administered to 9 neonatal Holstein calves at a dose of 1 mg/kg IV. Plasma samples were collected over 24 h and analyzed via HPLC-MS for determining pantoprazole concentrations. Pharmacokinetic parameters were derived via non-compartmental analysis. Tissue samples were collected at 1, 3, and 5 days after administration and analyzed via HPLC-MS.

**Results:** Following IV administration, plasma clearance, elimination half-life, and volume of distribution of pantoprazole were estimated at 4.46 mL/kg/min, 2.81 h, and 0.301 L/kg, respectively. The global extraction ratio was estimated at 0.053 ± 0.015. No pantoprazole was detected in the edible tissues 1, 3, or 5 days after administration. A metabolite, pantoprazole sulfone was detected in all the edible tissues 1 and 3 days after administration.

**Conclusion:** The reported plasma clearance for pantoprazole is less than that reported for alpacas but higher than reported in foals. The elimination half-life in calves appears to be longer than observed in foals and alpacas. While pantoprazole sulfone was detected in the tissues after IV administration, further research is needed as to the metabolism and potential tissue accumulation of other pantoprazole metabolites in calves. Future pharmacodynamic studies are necessary to determine the efficacy of pantoprazole on abomasal acid suppression in calves.

## Introduction

Abomasal ulceration is a multifactorial disease that is a common cause of morbidity and mortality throughout the beef and dairy industries. Reported prevalence of abomasal ulcers varies significantly owing to differences in the examined population, but ulcers can be found in cattle of all ages and management systems, with the highest prevalence in veal calves (1). Antemortem diagnosis is difficult as clinical signs are often subtle and can range from non-specific anorexia and abdominal pain to more obvious teeth grinding and melena. Abomasal ulceration can also contribute to peritonitis, which is a serious condition that can lead to sudden death (1–4). While the exact mechanism(s) leading to abomasal ulceration in ruminants are currently unknown, it can be assumed that the underlying cause is the disturbance of the equilibrium of protective and aggressive mechanisms on the gastric mucosa (5). Factors contributing to ulceration include age, weather, housing, stress, trauma, mineral deficiencies, bacterial overgrowth, and the use of non-steroidal anti-inflammatory drugs (NSAIDs) (2). Abomasal pH and gastric epithelial cellular damage caused by hydrochloric acid (HCl) secretion by parietal cells is also believed to play a role in ulcer formation (6).

Pantoprazole is a substituted benzimidazole that irreversibly binds to H+/K+ ATP pumps in gastric parietal cells to prevent the secretion of gastric acid (7). Pantoprazole is labeled in humans to effectively reduce acid secretion and increase gastric pH (8). In veterinary medicine, intravenous pantoprazole has also been demonstrated to effectively increase gastric pH in neonatal foals as well as adult alpacas (9, 10). In addition, subcutaneous administration in alpacas was shown to reach similar plasma levels as the intravenous administration and increase the pH of the third compartment (10). These studies in other large animal and production animal species, suggest the potential use of pantoprazole for the treatment of abomasal ulcers in ruminants. Acid neutralization, protection of the damaged mucosa, and prevention of acid secretion are amongst the accepted therapeutic interventions for gastric ulceration (11). Oral administration of antacids like aluminum or magnesium hydroxide can bind hydrochloric acid, absorb pepsin, and bind bile acids. These agents have been shown to cause a transient increase in abomasal pH of milk fed calves, but require oral administration, and absorption could be compromised by diseases such as ileus (12). Therefore, it could be more advantageous to use a parenterally administered drug, such as pantoprazole in hospitalized calves. Currently there are no labeled gastro-protectant products approved for use in food animals in the United States, so use of any drug is an extra-label manner.

The primary objective of this study was to determine the pharmacokinetic properties of a single intravenous (IV) administration of pantoprazole in neonatal calves. A secondary objective of this study was to look at tissue disposition after IV administration of pantoprazole.

## Materials and Methods

### Experimental Animals

This study was completed at the Iowa State University Food Animal and Camelid Hospital. Nine male Holstein calves were enrolled in the study. The age of these calves at enrollment was 2 days, the calves weighed 44.2 ± 4.3 kg and were procured from a single source farm. Approval for the study was secured from the Institution Animal Care and Use Committee (IACUC # 19-301) at Iowa State University. The calves were group housed in a climate-controlled environment. Criteria for enrollment in this study included a physical assessment by a veterinarian that yielded vital signs within the normal limits for a bovine calf, no previous history of medical illness as well as no history of a previously administered medication. Prior to and during the study, all calves were fed a diet that either met or exceeded the National Research Council (NRC) requirements for maintenance and growth of bovine calves. Study calves were fed a commercial milk replacer diet, with *ad libitum* access to water.

Twenty-four hours prior to initiation of the study, the calves were restrained and an IV jugular catheter was aseptically placed, and 2 h prior to the study a second IV jugular catheter was placed as previously described (13). The skin was aseptically prepared utilizing four alternating scrubs of chlorhexidine surgical scrub and 70% isopropyl alcohol. Prior to catheter placement, the skin at the catheter site was infiltrated with 2% lidocaine. The calf was restrained by study personnel and a 14-gauge catheter was placed in each jugular vein. An injection port was placed and the catheters were sutured to the skin and wrapped for security.

### Experimental Design and Sample Collection

Pantoprazole sodium (West-Ward, Eatontown, NJ, United States) was reconstituted to a 4 mg/mL concentration per manufacturer's recommendations and the calves were administered a single 1.0 mg/kg rapid IV bolus of pantoprazole via a catheter inserted in the left jugular vein. Blood collection was achieved through a catheter in the right jugular vein utilizing a previously described push-pull technique (14) at 0, 10, 20, 30, 45, 60, and 90 min as well as 2, 3, 4, 8, 12, 18, and 24 h after drug administration. Heart and respiratory rates, as well as rectal temperature were measured at time 0 and again at 24 h.

The pantoprazole dose (1.0 mg/kg) was determined based on a previous study investigating a similar dose in alpacas (10), as well as a retrospective study describing use of the drug in cattle, sheep, and goats (15). At each sampling time point, blood was collected from the catheter using a 12-mL syringe and placed into sodium heparin tubes (BD Vacutainer, Franklin Lakes, NJ). The samples were then centrifuged at 1,500 × g for 10 min. The plasma was pipetted off and transferred to cryovials which were then stored at −80°C until analysis.

### Plasma Sample Analysis

Plasma concentrations of pantoprazole were determined using ultra high-pressure liquid chromatography (UHPLC) with mass spectrometry detection after precipitation of plasma proteins with acetonitrile. The UHPLC consisted of an UltiMate 3,000 Pump, Column Compartment and Autosampler (Thermo Scientific, San Jose, CA, United States) coupled to an Orbitrap mass spectrometer (Q Exactive Focus, Thermo Scientific, San Jose, CA, United States). Bovine plasma samples were briefly thawed and centrifuged (2000 × g) prior to analysis. Plasma samples, plasma spikes, plasma QC's, and bovine plasma blanks, 50 μL, were added to 50 μL of water and then mixed with 400 μL of acetonitrile to precipitate plasma proteins. The acetonitrile contained pantoprazole-d3 (Toronto Research Chemicals, Ontario, Canada) as an internal standard at a concentration of 50 ng/mL. The samples were vortexed for 5 s and centrifuged for 10 min at 7,500 rpm (6,000 × g) to sediment the protein pellet. The supernatant was poured off into dry down tubes and evaporated at 40°C with a flow of nitrogen in a Turbovap. The contents were reconstituted with 200 μL of 25% acetonitrile in water. The samples were transferred to auto sampler vials fitted with a glass insert and centrifuged at 2,400 rpm (2,000 × g) prior to analysis.

Separation was achieved with a Hypersil Gold Vanquish column, 50 × 2.1 mm, 1.9 μm particles (Thermo Scientific, San Jose, CA, United States) maintained at 45°C. The analysis was performed starting at a solvent composition of 7.5% B which was increased linearly to 95% B in 4 min after injection. The solvent composition was maintained at 95% B for 1 min prior to equilibration to 7.5% B. The flow rate during this time period was 0.45 mL/min. Pantoprazole and pantoprazole-d3 eluted from the Hypersil Gold column at 3.38 ± 0.05 min. Parallel reaction monitoring in the positive electrospray ion mode was used for analyte detection. The precursor ions were determined by the instrument software from the molecular formulas. These were pantoprazole C16H15F2N3O4S or m/z of 384.082 and pantoprazole-d3 C16H12D3F2N3O4Sor m/z 384.101. Three or four fragment ions were used for quantitation of each analyte species. The fragment ions for pantoprazole were at 138.055, 153.078, and 200.037m/z, while ions at 138.055, 139.061, 156.097, and 203.056 m/z were characteristic of pantoprazole-d3 fragmentation.

Sequences consisting of plasma blanks, calibration spikes, quality control samples, and bovine plasma samples were analyzed and then batch processed with a processing method developed in the Xcalibur software (Thermo Scientific, San Jose, CA, United States). The processing method automatically identified and integrated each peak in each sample and calculated the internal standard based calibration curve using a weighted (1/X) linear fit. Plasma concentrations of pantoprazole in unknown samples were calculated by the Xcalibur software based on the calibration curve. Results were then viewed in the Quan Browser portion of the Xcalibur software. Twelve calibration spikes were prepared in blank bovine plasma covering the concentration range of 1.0 to 5,000 ng/mL. Calibration curves exhibited a correlation coefficient (r2) exceeding 0.999 across the concentration range. QC samples at 1.5, 15, 150, 750, and 3,000 ng/mL were within ± 15% of the nominal value with most of the QC's within ± 7.5% of the nominal value. Duplicate QC's at 150, 750, and 3,000 ng/mL were analyzed with each set of samples. The limit of quantitation (LOQ) of the analysis was 1.0 ng/mL with a lower limit of detection (LOD) of 0.2 ng/mL.

### Tissue Sample Collection

The calves were euthanized in groups of three, on days 1, 3, and 5 post pantoprazole administration. All calves were euthanized using a penetrating captive bolt followed by an injectable potassium chloride solution per the American Veterinary Medical Association's euthanasia guidelines (16). Samples of kidney, liver, fat, and muscle were collected from the calves immediately after euthanasia and were then stored at −80°C until analysis.

### Tissue Sample Analysis

Tissue concentrations of pantoprazole were determined using ultra high-pressure liquid chromatography (UHPLC) with mass spectrometry detection. The UHPLC consisted of an UltiMate 3,000 Pump, Column Compartment and Autosampler (Thermo Scientific, San Jose, CA, United States) coupled to an Orbitrap mass spectrometer (Q Exactive Focus, Thermo Scientific, San Jose, CA, United States). The tissue samples analyzed were muscle, kidney, liver, and fat. Tissue samples were thawed and homogenized in a Waring blender prior to extraction and analysis. Tissue samples, tissue spikes, and blanks, 2 grams of tissue homogenate, were extracted with 20 mL of a 4:1 mixture of acetonitrile:water in a 50 mL centrifuge tube. Tissue samples from calves of similar age, with no history of drug administration were used as blanks for control. An internal standard, d3-pantoprazole, was added to the tissue homogenate, prior to extraction with 20 μL of a 50 ng/μL solution. The solvent extraction was performed on a multi-tube vortex mixer at 2,000 rpm for 15 min after the addition of the acetonitrile mixture. Subsequently the extracted samples were centrifuged for 5 min at 3,000 rpm and filtered thru glass fiber filters into 15 mL centrifuge tubes. A portion (500 μL) of each extract was diluted with 1,000 μL of water and samples were centrifuged at 2,500 rpm prior to LC-MS/MS analysis.

For LC-MS/MS analysis the injection volume was set to 2.0 μL. The mobile phases consisted of A: 0.1% formic acid in water and B: 0.1% formic acid in methanol at a flow rate of 0.35 mL/min. The mobile phase began at 7.5% B with a linear gradient to 95% B in 4.0 min, which was maintained for 1 min at 0.45 mL/min, followed by re-equilibration to 7.5% B also at 0.45 mL/min. Separation was achieved with a HypersilGoldC18 column, 50 x 2.1 mm, 1.9 μm particles, Thermo Scientific, San Jose, CA, United States) maintained at 45°C. Pantoprazole and d3-pantoprazole each eluted at 3.37 ± 0.02 min. Parallel reaction monitoring in the positive electrospray ion mode was used for analyte detection. The precursor ions were determined by the instrument software from the molecular formulas. These were pantoprazole C16H15F2N3O4S or m/z of 384.082 and d3-pantoprazole C16H12D2F2N3O4S or m/z 387.101. Three or four fragment ions were used for quantitation of each analyte species. The fragment ions for pantoprazole were at 138.05, 153.08, and 200.04 m/z, while ions at 138.05, 139.06, 156.09, and 203.06 m/z were characteristic of d3-pantoprazole fragmentation. Sequences consisting of tissue blanks, calibration spikes, quality control samples, and bovine tissue samples were batch processed with a processing method developed in the Xcalibur software (Thermo Scientific, San Jose, CA, United States). The processing method automatically identified and integrated each peak in each sample and calculated the calibration curve based on a weighted (1/X) linear fit. Nine calibration spikes were prepared in blank bovine tissue covering the concentration range of 0.01 to 10 μg/g. Three QC samples were also prepared in each tissue matrix at 0.075, 0.30, and 3.0 μg/g. Tissue concentrations of pantoprazole and in unknown samples were calculated by the Xcalibur software based on the calibration curve. Results were then viewed in the Quan Browser portion of the Xcalibur software. Calibration curves exhibited a correlation coefficient (*r*^2^) exceeding 0.997 across the concentration range. All QC samples in each tissue matrix were within the ± 15 % criteria for acceptability with the majority being within ± 5 % of the nominal value. The limit of quantitation (LOQ) of the pantoprazole analysis was 0.01 ug/g with a limit of detection (LOD) of 0.002 ug/g.

Fifteen metabolites of pantoprazole were screened for in most of the kidney and liver extracts. All these metabolites were screened in full scan mode on the Q Exactive instrument. These metabolites were divided into three groups. The first group consisted of 7 metabolites arising thru glutathione conjugation of pantoprazole and are identified as M1-M6 and M8 by 16 Zhong et al. (17) Another group consisted of pantoprazole sulfone, pantoprazole sulfide, and 4'-O-demethylpantoprazole. These three potential metabolites are at m/z of 400.077, 368.087, and 370.067 resulting from the addition and loss of an oxygen from pantoprazole for the first two metabolites and loss of a methylene group for the third potential metabolite. These three potential metabolites were screened by full scan mode and parallel reaction monitoring. The last set of metabolites consisted of 4'-O-demethylpantoprazole sulfide, 4'-O-demethylpantoprazole sulfone, Pantoprazole sulfone N-oxide, Pantoprazole N-oxide, and Hydroxy pantoprazole. The last two metabolites share the same exact mass of 400.077 with pantoprazole sulfone. The potential metabolites of the first group, M1-M6 & M8, were not present in any of the liver or kidney extracts. A consistent, moderate intensity, chromatographic peak was obtained at the 400.077 mass. This peak was not present in extracts of tissue controls spiked with pantoprazole. This peak could consist of one of three metabolites; pantoprazole sulfone, pantoprazole N-oxide, or Hydroxy pantoprazole. Chromatograms of the potential metabolites are shown in Supplemental Figures 1 and 2.

The mass spectra and retention time of the peak at 3.45 min in Supplemental Figure 3 matched that of a standard of pantoprazole sulfone. Therefore, all the tissue samples were re-extracted as outlined previously in our methods. Pantoprazole sulfone (Toronto Research Chemicals, Ontario, Canada) became the analyte spiked into blank tissue samples to generate calibration spikes and QC samples. Parallel reaction monitoring in the positive electrospray ion mode was used for analyte detection. The precursor ion of pantoprazole sulfone, C16H15F2N3O5S or m/z of 400.077 was detected by parallel reaction monitoring. Four fragment ions were used for quantitation of pantoprazole sulfone were at 122.06, 152.07, 168.07, and 336.12 m/z. The calibration range for pantoprazole sulfone was 0.001 to 5 μg/g in each tissue. Three QC samples were also prepared in each tissue matrix at 0.075, 0.30, and 3.0 μg/g. Calibration curves exhibited a correlation coefficient (*r*^2^) exceeding 0.997 across the concentration range. All QC samples in each tissue matrix were within the ± 15 % criteria for acceptability with the majority being within ± 5 % of the nominal value. The limit of quantitation (LOQ) of the pantoprazole analysis was 0.002 μg/g with a limit of detection (LOD) of 0.0005 μg/g.

### Pharmacokinetic Analysis

Pharmacokinetic parameters were then determined from plasma concentration data. Pharmacokinetic modeling was performed via standard industry modeling software (PKanalix, Monolix Suite 2020R1, Lixoft, France). Standard time vs. concentration data for pantoprazole were determined via liquid chromatography-mass spectrometry from the blood collected at 14 time points ranging from 0 to 24 h after administration.

Standard PK parameters were generated for individual calves, as follows:

1. Maximum concentration extrapolated to time zero, C_0_ (pantoprazole);
2. Maximum pantoprazole concentration, C_max_ (pantoprazole);
3. Area under pantoprazole concentration–time curve, AUC_last_ and AUC_inf_;
4. Area under the moment curve, AUMC_inf_;
5. Pantoprazole mean residence time, MRT = AUMC_inf_/AUC_inf_;
6. Pantoprazole terminal half-life, T_1/2_ (λz)) = ln (2)/λz;
7. Pantoprazole systemic clearance, CL = Dose/AUC_inf_;
8. Volume of distribution of pantoprazole at steady-state, V_ss_ = CL × MRT.

For data analysis, a linear/log trapezoidal rule was used to estimate the area under the pantoprazole time-curves. Summary statistics on the individual PK parameters were performed thereafter to derive the geometric mean, median and (min–max) range.

The global extraction ratio (E_body_) was calculated as reported by Toutain and Bousquet-Melou (18), with:

E_body_ =Systemic clearance/Cardiac output

First calculated for each individual calf, and then combined for a mean value. With the calf cardiac output calculated as previously reported (13, 18) as follows:

Cardiac output=180 × BW(kg)^−0.19^.

## Results

### Animal Health

All the animals in the study were deemed healthy and had vital parameters within the normal limits for neonatal calves at the time of enrollment. Intravenous catheter placement was well-tolerated and no signs of thrombophlebitis were observed during the course of the study. Administration of pantoprazole was handled without any visual observations of clinical manifestations of adverse effects.

### Pharmacokinetics

**Figure 1:**
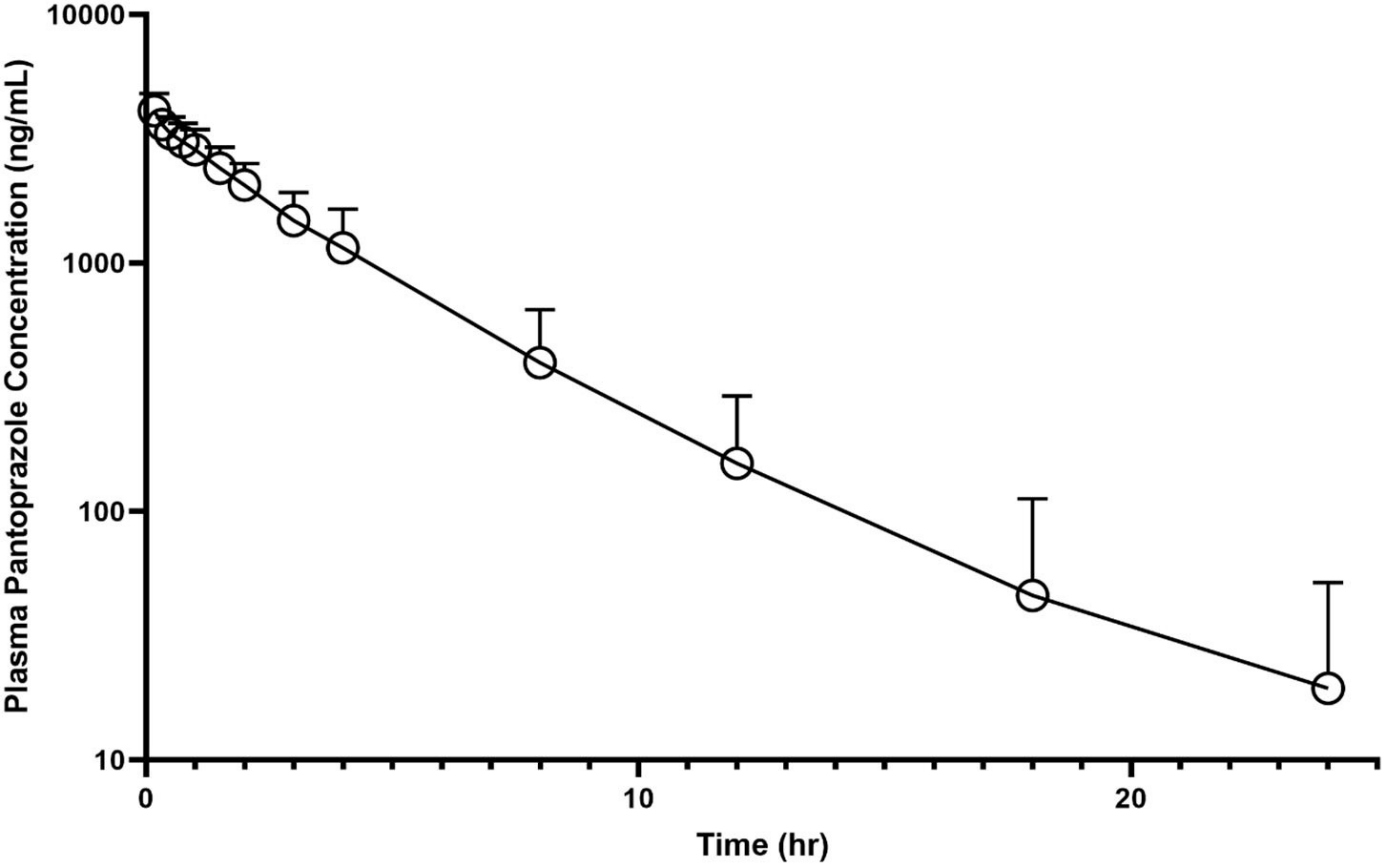
Mean plasma pantoprazole concentration (logarithmic scale) vs. time (hr) profiles for neonatal calves (*n* = 9) following intravenous (IV) single dose administration of 1.0 mg/kg of pantoprazole.

No calf had detectable pantoprazole in plasma at time zero. The individual time-course of pantoprazole total concentrations in plasma can be found in Figure 1. Among individual calves, there appears to be limited variation of time vs. concentration data for pantoprazole.

Table 1 summarizes the pharmacokinetic parameters for pantoprazole when administered IV. The systemic clearance was 4.46 mL kg^−1^ min^−1^. The AUC% extrapolation was estimated to be inferior to 20% (0.24%), while the steady-state volume of distribution (V_ss_) was 0.257 L/kg. The plasma elimination half-life T1/2 (λz) was estimated at ~2.8 h. The average extraction ratio was calculated to be 0.053 ± 0.015.

**Table 1:** Pantoprazole pharmacokinetic parameters following a single intravenous (1 mg/kg) administration to neonatal Holstein calves.

**Compound**	**Parameter**	**Unit**	**Geomean**	**Median**	**Min**	**Max**
Pantoprazole	AUC_last_	hr*ug/mL	13.37	12.65	8.56	25.44
	AUC_inf_	hr*ug/mL	13.44	12.66	8.57	26.18
	AUMC_inf_	hr*ug/mL	46.41	49.66	20.77	153.16
	MRT_inf_	hr	3.45	3.52	2.42	5.85
	Cl	mL/kg/min	4.46	4.74	2.29	7.02
	T_1/2_ (λz)	hr	2.81	2.72	1.98	5.16
	C0	ug/mL	4.62	4.11	3.83	7.48
	C_max_	ug/mL	4.07	3.90	3.57	5.82
	V_z_	L/kg	0.301	0.285	0.239	0.421
	V_ss_	L/kg	0.257	0.261	0.224	0.327

*AUC_last_, Area under pantoprazole concentration–time curve from time zero to last measurement; AUC_inf_, Area under pantoprazole concentration–time curve from time zero to infinity; AUMC_inf_, the area under the first-moment curve from time zero to infinity; MRT_inf_, Mean residence time extrapolated to infinity; Cl, Plasma clearance; T_1/2_ (λz), Elimination half-life; C0, Maximum concentration extrapolated to time zero; C_max_, Maximum concentration; V_z_, Volume of distribution (terminal phase); V_ss_, Volume of distribution at steady-state*.

### Tissue Concentrations

No concentrations of pantoprazole were detected in muscle, liver, kidney or fat from any calves at 1, 3, or 5 days after administration. Analysis of the tissues for pantoprazole sulfone, a metabolite of pantoprazole, revealed detectable levels in all tissues in calves 1 and 3 days post-administration. One day after administration, the highest concentrations of pantoprazole sulfone were found in kidney, followed by liver, then fat, with muscle having the lowest concentration of the metabolite. Three days after administration revealed the same distribution. Five days after administration, the highest concentrations of pantoprazole sulfone were found in kidney, followed by liver, then the muscle. The level of pantoprazole sulfone in the fat 5 days post administration was below our limit of detection in all three calves. For our calves sacrificed at day five post administration, one calf was below the limit of detection in all tissues, one was below the limit for muscle and fat, and one was below the limit for fat. Table 2 gives a summary of these findings.

**Table 2:** Tissue concentrations of pantoprazole sulfone (μg/g) in collected tissues 1, 3, and 5 days after intravenous administration of pantoprazole (1 mg/kg) from study calves.

**Days post dose**	**Animal ID**	**Liver (μg/g)**	**Kidney (μg/g)**	**Muscle (μg/g)**	**Fat (μg/g)**
1	2	0.035	0.045	0.011	0.014
	7	0.111	0.120	0.039	0.043
	8	0.056	0.069	0.027	0.034
3	5	0.018	0.014	0.009	0.010
	6	0.029	0.038	0.016	0.022
	9	0.005	0.006	0.003	0.003
5	1	0.003	0.004	0.001	< LOQ
	3	0.002	0.008	< LOQ	< LOQ
	4	< LOQ	< LOQ	< LOQ	< LOQ

*<LOQ: concentration below the lower limit of quantification. 1 μg/g = 1 part per million (PPM)*.

## Discussion

To our knowledge, this is the first report of pharmacokinetics of pantoprazole in neonatal calves. Although the cohort sampling could potentially be a source of bias for this study, it was thought to be minimal as calves had acclimated to the pen prior to the study, and the pen used for the study was from the same block of stalls in the temperature, humidity and ventilation-controlled barn. The age and size of the calves utilized for this study were similar in the age of calves presented to the author's hospital for medical treatment that could potentially benefit from pantoprazole.

In the United States, there are currently no approved parenterally administered gastroprotectants for food animal species. However, patients in the author's hospital are commonly prescribed such drugs during hospitalization. There are two major classes of parenterally administered gastroprotectants commonly used in large animal species: histamine-2 receptor antagonists (H2RA) and proton pump inhibitors (PPI). H2RAs, such as ranitidine and famotidine, decrease the production of gastric acid by binding to H2 receptors on parietal cells (19). These drugs can be administered intravenously in cattle, however a recent study showed that famotidine only had a transient effect on abomasal pH that decreased with subsequent doses (20). H2RA also require multiple daily administrations (20), which could lead to treatment challenges. Omeprazole is a PPI similar to pantoprazole that has been shown to reduce abomasal pH in milk fed calves though subsequent doses may have a reduced effect (21). Intravenous omeprazole has been shown effective in reducing gastric pH in a variety of species however the intravenous formulation is no longer available, or has very limited availability for food animal veterinary use. Pantoprazole is also a PPI with similar structure and properties to omeprazole that is frequently used in human health as a gastroprotectant. Other studies have evaluated the pharmacokinetics of intravenous pantoprazole in foals (9) and alpacas (10). Comparatively, the AUC_inf_ of the calves in our study (13.44 hr^*^μg/ml) falls in between that of alpacas (1.42 hr^*^ μg /ml) and foals (18.7 hr^*^ μg/ml). These comparisons can be found in Table 3. The clearance of pantoprazole in calves appears to be much lower than alpacas but greater than foals. Additionally, in our calves a longer half-life was noted when compared to alpacas and foals.

**Table 3:** Comparisons of pharmacokinetic parameters of pantoprazole in domestic animal species, after single dose intravenous administration.

**Parameter (Units)**	**Calves (present study)**	**Alpacas(10)**	**Foals(9)**
Dose (mg/kg)	1	1	1.5
AUC_inf_ (hr*ug/ml)	13.44	1.42	18.7
Cl (mL/kg/min)	4.46	12.2	1.34
C_max_ (ug/mL)	4.07	N/A	4.08
T_½_ (λz) (hr)	2.81	0.47	1.43
V_ss_ (L/kg)	0.257	0.49	N/A
MRT (h)	3.45	0.68	2.13
LLOQ (ng/mL)	0.2	10	200

*AUC_inf_, Area under pantoprazole concentration–time curve from time zero to infinity; Cl, Plasma clearance; C_max_: Maximum concentration; T_1/2_ (λz), Elimination half-life; V_ss_, Volume of distribution at steady-state; MRT: Mean residence time; C0: LLOQ: Lower limit of quantification*.

When comparing pharmacokinetic parameters between our study and that of the alpaca and foal studies, it is important to note the differences in the lower limit of detection of quantification (LLOQ). The LLOQ for our current study is 0.2 ng/ml which is a considerably smaller value when compared to the LLOQ of the alpaca study (10 ng/mL) and that of the foal study (200 ng/mL). As such, our study was able to detect lower quantities of pantoprazole than the alpaca or foal studies and therefore could find detectable levels of pantoprazole for a longer period of time compared to those studies. This may extend the half-life from our study when comparing to the other studies. This has been noted for other drugs in calves, an example being the comparison of the pharmacokinetics of fentanyl in calves to studies with higher LLOQ in goats and alpacas (13).

Although some cases of post pantoprazole administration anaphylaxis (22, 23) and edema (24, 25) have been reported in humans, these clinical signs were not observed in our population. While the aim of this study was not to evaluate the clinical safety of pantoprazole in calves, the lack of observed adverse effects correlate with previously reported pantoprazole use in ruminants for case management of cattle (4, 26), camels (27), sheep (15, 28), yaks (29), and goats (30, 31). Adverse effects of pantoprazole administration described people include hyponatremia (32), hypomagnesemia (33), as well as nephritis and hepatotoxicity (34). Additional adverse effects described in people from proton pump inhibitor administration include thrombocytopenia (35) and neutropenia (36).

The parent form of pantoprazole was not detected in any of the tissue samples submitted, however we were able to identify the sulfone metabolite in the tissues. Based on previous studies in rats and humans, it appears that pantoprazole is extensively metabolized by the liver into its metabolites (16). Our data appears to reflect a similar rapid metabolism, and it is possible that metabolism of pantoprazole is similar in ruminants as in rats. However, these details are currently unknown. Based on these findings, residue avoidance recommendations may want to be made considering the sulfone metabolite, instead of the parent compound. Further studies will be necessary to completely elucidate the complete metabolism of pantoprazole in cattle.

Limitations of this study are the relatively small number of calves used, which might not account for the variability of pantoprazole pharmacokinetics in the population of calves. Similarly, all of the animals were neonatal calves of the approximate same age and from the same source farm, which may not be reflective of all cattle. All animals in this study were deemed healthy which also may not represent the cohort of patients receiving this drug. While our study did not report any adverse effect, it was not designed as a safety study. A retrospective study has suggested that pantoprazole may be safe to administer to hospitalized ruminants (15). Future prospective studies will be necessary to determine the clinical safety as well as the efficacy of pantoprazole in cattle. Further studies could also investigate the concentrations of pantoprazole at other tissues, such as the abomasum, with larger cohorts of calves to further explore tissue terminal elimination rates.

In summary, this study reports the pharmacokinetics and tissue concentrations of intravenously administered pantoprazole to calves. The plasma clearance of pantoprazole in calves is lower than that of alpacas, and higher than reported in foals. The plasma elimination half-life in calves is longer than reported in those species. Currently, pantoprazole is being used in an extra-label fashion in food animal species, therefore further investigations into its metabolites and tissue concentrations with larger numbers of animals are necessary to establish withdrawal periods. Future pharmacodynamic studies are needed to determine if plasma values correlate to clinical efficacy in the form of increased abomasal pH in cattle of various ages.

## Data Availability Statement

The raw data supporting the conclusions of this article will be made available by the authors, without undue reservation.

## Ethics Statement

The animal study was reviewed and approved by Institutional Animal Care and Use Committee, Iowa State University.

## Author Contributions

JO, AK, DT, KD, JM, and JS conceptualized the plan of work. LW developed the analytical method for plasma and tissue concentration determination. All authors contributed to manuscript construction.

## Conflict of Interest

The authors declare that the research was conducted in the absence of any commercial or financial relationships that could be construed as a potential conflict of interest.

## References

[1] Hund A Wittek T. Abomasal and third compartment ulcers in ruminants and south American camelids. Vet Clin North Am Food Anim Pract. (2018) 34:35–54. 10.1016/j.cvfa.2017.10.003 29276097

[2] Marshall T. Abomasal ulceration and tympany of calves. Vet Clin N Am Food Anim Pract. (2009) 25:209–20. 10.1016/j.cvfa.2008.10.010 19174290

[3] Smith DF Munson L Erb HN. Predictive values for clinical signs of abomasal ulcer disease in adult dairy cattle. Prev Vet Med. (1986) 3:573–80. 10.1016/0167-5877(86)90035-8 6884033

[4] Guarnieri E Fecteau G Berman J Desrochers A Babkine M Nichols S. Abomasitis in calves: a retrospective cohort study of 23 cases (2006-2016). J Vet Intern Med. (2020) 34:1018–27. 10.1111/jvim.15726 32056284 PMC7096666

[5] Smith BP Van Metre D Pusterla N Large Animal Internal Medicine. Smith BP Van Metre D Pusterla N, editors. 6th Edn St. Louis, Missouri: Elsevier (2020)

[6] Laine L Takeuchi K Tarnawski A. Gastric mucosal defense and cytoprotection: bench to bedside. Gastroenterology. (2008) 135:41–60. 10.1053/j.gastro.2008.05.030 18549814

[7] Cheer SM Prakash A Faulds D Lamb HM Pantoprazole - an update of its pharmacological properties and therapeutic use in the management of acid-related disorders. Drugs. (2003) 63:101–32. 10.2165/00003495-200363010-00006 12487624

[8] Dekel R Morse C Fass R. The role of proton pump inhibitors in gastro-oesophageal reflux disease. Drugs. (2004) 64:277–95. 10.2165/00003495-200464030-00004 14871170

[9] Ryan CA Sanchez LC Giguere S Vickroy T. Pharmacokinetics and pharmacodynamics of pantoprazole in clinically normal neonatal foals. Equine Vet J. (2005) 37:336–41. 10.2746/0425164054529427 16028623

[10] Smith GW Davis JL Smith SM Gerard MP Campbell NB Foster DM. Efficacy and pharmacokinetics of pantoprazole in alpacas. J Vet Intern Med. (2010) 24:949–55. 10.1111/j.1939-1676.2010.0508.x 20384953

[11] Marks SL Kook PH Papich MG Tolbert MK Willard MD. ACVIM consensus statement: support for rational administration of gastrointestinal protectants to dogs and cats. J Vet Intern Med. (2018) 32:1823–40. 10.1111/jvim.15337 30378711 PMC6271318

[12] Ahmed AE Constable PD Misk N. Effect of an orally administered antacid agent containing aluminum hydroxide and magnesium hydroxide on abomasal luminal pH in clinically normal milk-fed calves. J Am Vet Med Assoc. (2002) 220:74–9. 12680452 10.2460/javma.2002.220.74

[13] Smith JS Coetzee JF Fisher IWG Borts DJ Mochel JP. Pharmacokinetics of fentanyl citrate and norfentanyl in holstein calves and effect of analytical performances on fentanyl parameter estimation. J Vet Pharmacol Ther. (2018) 41:555–61. 10.1111/jvp.12501 29603262

[14] Barr CA Gianotti G Graffeo CE Drobatz KJ Silverstein DC Effect of blood collection by the push-pull technique from an indwelling catheter versus direct venipuncture on venous blood gas values before and after administration of alfaxalone or propofol in dogs. J Am Vet Med Assoc. (2017) 251:1166–74. 10.2460/javma.251.10.1166 29099261

[15] Smith JS Kosusnik AR Mochel JP. A retrospective clinical investigation of the safety and adverse effects of pantoprazole in hospitalized ruminants. Front Vet Sci. (2020) 7:97. 10.3389/fvets.2020.00097 32258063 PMC7089877

[16] Association APoEAVM 2000 Report of the AVMA panel on euthanasia. J Am Vet Med Assoc. (2001) 218:669–96. 10.2460/javma.2001.218.669 11280396

[17] Zhong D Xie Z Chen X. Metabolism of pantoprazole involving conjugation with glutathione in rats. J Pharm Pharmacol. (2005) 57:341–9. 10.1211/0022357055669 15807990

[18] Toutain PL Bousquet-Melou A Plasma clearance. J Vet Pharmacol Ther. (2004) 27:415–25. 10.1111/j.1365-2885.2004.00605.x 15601437

[19] Berlin RG Clineschmidt BV Majka JA. Famotidine: an appraisal of its mode of action and safety. Am J Med. (1986) 81:8–12. 10.1016/0002-9343(86)90594-2 2877577

[20] Balcomb CC Heller MC Chigerwe M Knych HK Meyer AM. Pharmacokinetics and efficacy of intravenous famotidine in adult cattle. J Vet Intern Med. (2018) 32:1283–9. 10.1111/jvim.15080 29572958 PMC5980459

[21] Ahmed AF Constable PD Misk NA. Effect of orally administered omeprazole on abomasal luminal pH in dairy calves fed milk replacer. J Vet Med A Physiol Pathol Clin Med. (2005) 52:238–43. 10.1111/j.1439-0442.2005.00715.x 15943608

[22] Gupta PP Bhandari R Mishra DR Agrawal KK Jirel S Malla G. Anaphylactic reactions due to pantoprazole: case report of two cases. Int Med Case Rep J. (2018) 11:125–7. 10.2147/IMCRJ.S153099 29872353 PMC5973311

[23] Natsch S Vinks MH Voogt AK Mees EB Meyboom RH. Anaphylactic reactions to proton-pump inhibitors. Ann Pharmacother. (2000) 34:474–6. 10.1345/aph.19235 10772433

[24] Sennaroglu E Karakan S Kayatas M Akdur S Genc H Karakan T. Reversible edema in a male patient taking parenteral pantoprazole infusion for pyloric stenosis. Dig Dis Sci. (2006) 51:121–2. 10.1007/s10620-006-3095-1 16416223

[25] Brunner G Athmann C Boldt JH. Reversible pheripheral edema in female patients taking proton pump inhibitors for peptic acid diseases. Dig Dis Sci. (2001) 46:993–6. 10.1023/A:1010745624971 11341670

[26] Smith JS Zhou X Merkatoris PT Klostermann CA Breuer RM. Medical management of hemorrhagic bowel syndrome in a beef bull. Case Rep Vet Med. (2019) 2019:9209705. 10.1155/2019/9209705 31781470 PMC6875306

[27] Rosser JM Jacob SI Brounts SH. Use of tube cystostomy in the surgical management of obstructive urolithiasis in a bactrian camel. J Am Vet Med Assoc. (2019) 254:868–73. 10.2460/javma.254.7.868 30888274

[28] Viall AK Larios Mora A Brewer MT Smith JS Kreuder AJ Breuer RM. What is your diagnosis? Nasal discharge from a sheep. Vet Clin Pathol. (2018) 47:503–4. 10.1111/vcp.12613 29733440

[29] Smith JS Sheley M Chigerwe M. Aspiration pneumonia in two tibetan yak bulls (Bos grunniens) as a complication of ketamine-xylazine-butorphanol anesthesia for recumbent castration. J Zoo Wildlife Med. (2018) 49:242–6. 10.1638/2016-0205R1.1 29517446

[30] Smith J Klostermann C Harm T Breuer R Kovalik DA Bornkamp J Abomasal hamartoma in a la mancha wether. Vet Rec Case Rep. (2017) 5:e000515 10.1136/vetreccr-2017-000515

[31] Poirier NC Smith JS Breuer RM Farrell AM Klostermann CA Tseng CT Management of hematometra in a boer doe. Clin Theriogenol. (2020) 12:39–45.

[32] Falhammar H Lindh JD Calissendorff J Skov J Nathanson D Mannheimer B. Associations of proton pump inhibitors and hospitalization due to hyponatremia: a population-based case-control study. Eur J Intern Med. (2019) 59:65–9. 10.1016/j.ejim.2018.08.012 30154038

[33] Liao S Gan L Mei Z. Does the use of proton pump inhibitors increase the risk of hypomagnesemia: an updated systematic review and meta-analysis. Medicine. (2019) 98:e15011. 10.1097/MD.0000000000015011 30921222 PMC6456119

[34] Aslan M Celik Y Karadas S Olmez S Cifci A. Liver hepatotoxicity associated with pantoprazole: a rare case report. Wien Klin Wochenschr. (2014) 126:390–2. 10.1007/s00508-014-0535-3 24652021

[35] Kallam A Singla A Silberstein P. Proton pump induced thrombocytopenia: a case report and review of literature. Platelets. (2015) 26:598–601. 10.3109/09537104.2014.953045 25207666

[36] Yu Z Hu J Hu Y. Neutropenia and thrombocytopenia induced by proton pump inhibitors: a case report. Drug Saf Case Rep. (2018) 5:28. 10.1007/s40800-018-0093-0 30470925 PMC6251937

