# Supplementary material for: Pharmacokinetics and Tissue Levels of Pantoprazole in Neonatal Calves After Intravenous Administration
Source: Front Vet Sci. 2020 Nov 27;7:580735. doi: 10.3389/fvets.2020.580735 (PMC7728716; doi:10.3389/fvets.2020.580735)
Supplement: Supplementary file 1 [file Table_1.pdf]

Supplementary Material

1.1 Supplementary Figures

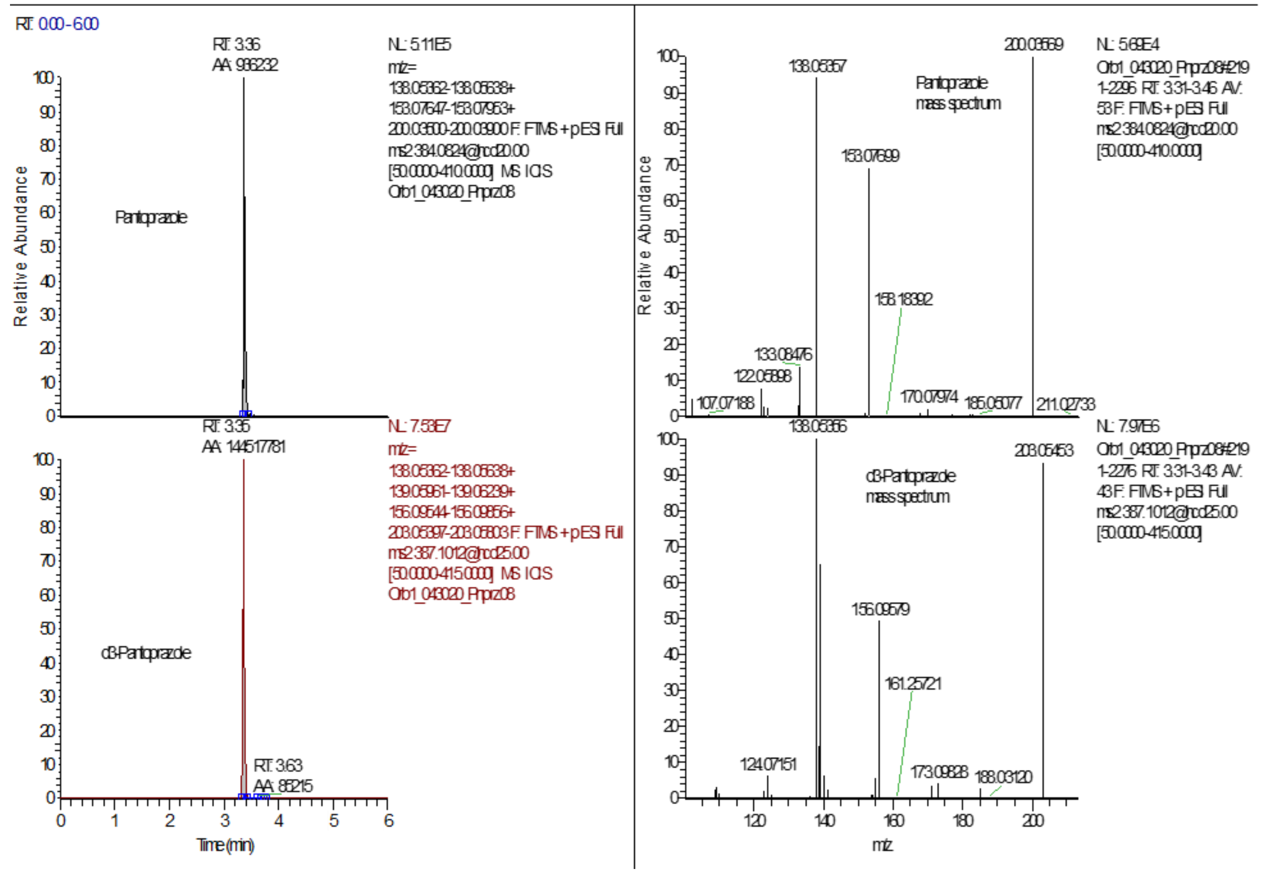

Supplementary Figure 1. Chromatogram and ion fragments of Pantoprazole in a Kidney Tissue Spike

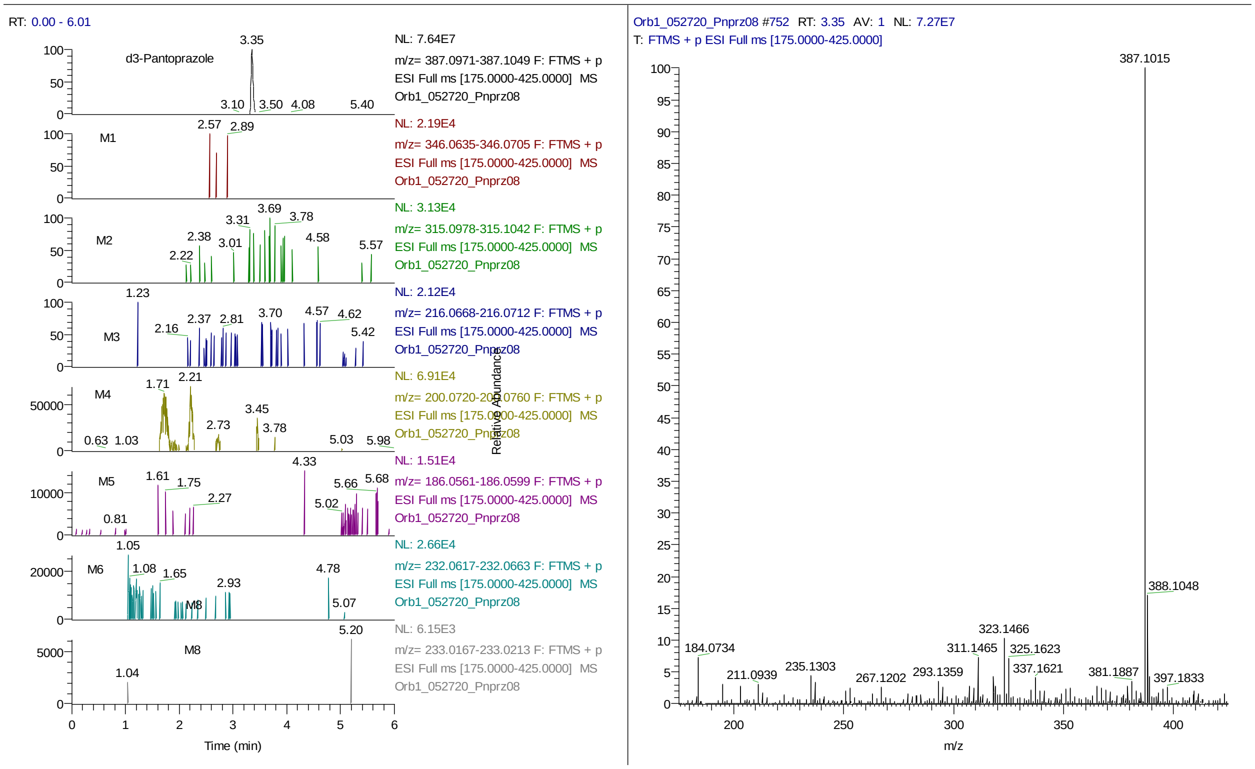

Supplementary Figure 2. Chromatogram of potential metabolites M1-M6 & M8 in a liver sample

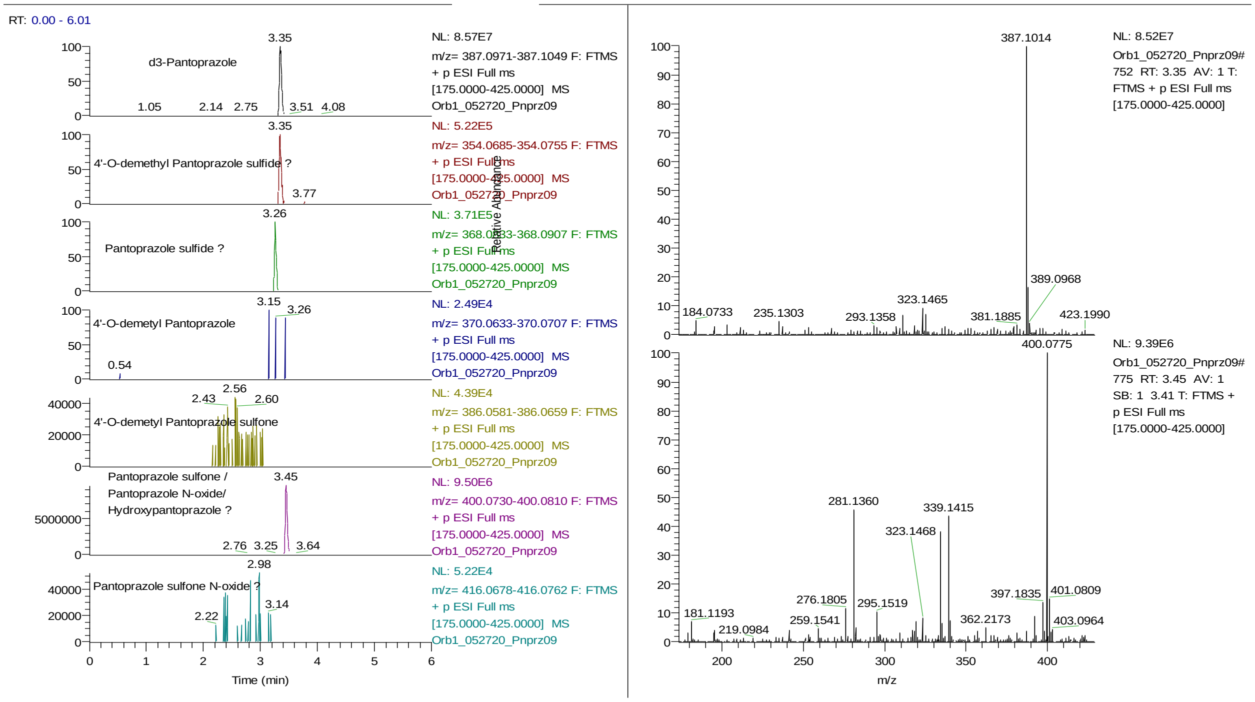

Supplementary Figure 3. Chromatogram of potential metabolites in a liver sample
